# Preconditioning-induced ischemic tolerance: a window into endogenous gearing for cerebroprotection

**DOI:** 10.1186/2040-7378-2-2

**Published:** 2010-01-21

**Authors:** Aysan Durukan, Turgut Tatlisumak

**Affiliations:** 1 Department of Neurology, Helsinki University Central Hospital, Helsinki, Finland

## Abstract

Ischemic tolerance defines transient resistance to lethal ischemia gained by a prior sublethal noxious stimulus (i.e., preconditioning). This adaptive response is thought to be an evolutionarily conserved defense mechanism, observed in a wide variety of species. Preconditioning confers ischemic tolerance if not in all, in most organ systems, including the heart, kidney, liver, and small intestine. Since the first landmark experimental demonstration of ischemic tolerance in the gerbil brain in early 1990's, basic scientific knowledge on the mechanisms of cerebral ischemic tolerance increased substantially. Various noxious stimuli can precondition the brain, presumably through a common mechanism, genomic reprogramming. Ischemic tolerance occurs in two temporally distinct windows. Early tolerance can be achieved within minutes, but wanes also rapidly, within hours. Delayed tolerance develops in hours and lasts for days. The main mechanism involved in early tolerance is adaptation of membrane receptors, whereas gene activation with subsequent *de novo *protein synthesis dominates delayed tolerance. Ischemic preconditioning is associated with robust cerebroprotection in animals. In humans, transient ischemic attacks may be the clinical correlate of preconditioning leading to ischemic tolerance. Mimicking the mechanisms of this unique endogenous protection process is therefore a potential strategy for stroke prevention. Perhaps new remedies for stroke are very close, right in our cells.

## Review

Surviving a sublethal noxious insult may result in a more powerful state against a following lethal insult, referring to Nietszche; "What doesn't kill you, makes you stronger." This phenomenon named as preconditioning (PC) and tolerance has been shown to exist in many organs, most extensively in the heart. The first in vivo evidence of preconditioning and tolerance in brain was provided in 1960's [1,2], but almost three decades passed without any interest from researchers on this unique phenomenon, until Kitagawa et al. opened the research era of cerebral ischemic tolerance (IT) [3].

**Figure 1:**
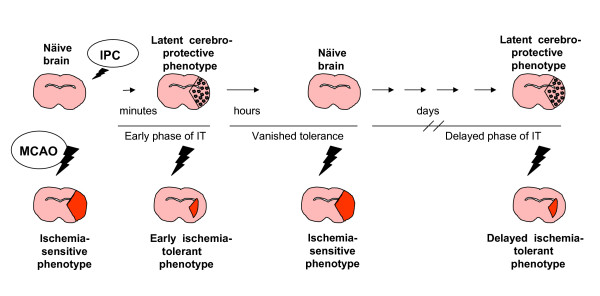
**Outcomes in the different settings with middle cerebral artery occlusion (MCAO)**. In the absence of ischemic preconditioning (IPC), MCAO induces a large infarction (ischemia-sensitive phenotype). IPC results early ischemic tolerance (IT) in minutes; if MCAO is applied during this phase, mainly cortical areas are spared (early ischemia-tolerant phenotype). In hours, the brain regresses to its naïve state. Delayed phase of IT occurs in days, when the latent cerebroprotective phenotype is complete, the brain is again ischemia-tolerant.

The ability to withstand, respond to, and to cope with ongoing stress is a fundamental property of all living organisms [4]. The fate of the brain tissue after focal cerebral ischemia is determined by the degree and duration of ischemia, and even without preconditioning, resident brain cells naturally respond to brain ischemia by mobilizing a host of defences and counter responses to mitigate cell injury and death [5]. If the subthreshold noxious stimulus is too mild or negligibly mild, it may not induce any response, whereas if it is sufficient enough, it may serve as a PC trigger, or if it is too severe, over the threshold, may permanently injure tissues. The hallmark of PC stimulus is not being injurious. In the scenario of IT, PC stimulus primes the brain for subsequent injurious ischemic injury. Danger signal evoked in the brain by the stressing preconditioning stimulus induces complex endogenous protective mechanisms resulting to a latent protective phenotype. When the lethal ischemic insult is applied onto this latent protective phenotype, a separate set of responses are triggered that constitute ischemia-tolerant phenotype, which strikingly differs from the unprimed or unpreconditioned brain's phenotype (Figure 1). Therefore, the outcome of the brain cells is shifted by PC from death to survival.

During the last years, the mechanisms underlying cerebral IT were intensely studied, and although incomplete, a vast amount of knowledge has been accumulated. The salient features of cerebral IT are presented in Table 1. There are two temporally distinct windows of protection from ischemia afforded by PC. Early protection, i.e., early IT, has been observed in relatively fewer studies than those exposing late IT in the brain. Exploring the functional relevance of these findings has proved difficult, however.

**Table 1:** Main futures of cerebral ischemic tolerance

General	Preconditioning specific
• Robust cerebroprotection	• Two phased: early and delayed
• The interval between preconditioning and ischemia determines the fate	• Early tolerance starts in minutes, delayed tolerance not usually before 24 h
• Ischemic tolerance is transient	• After early phase, but before delayed phase no tolerance is achieved
• Ischemic tolerance can be induced by a variety of stimuli	• Early phase is short-lasting, delayed phase longer, up to 1 week
• Transient ischemic attacks confer ischemic tolerance in humans	• Preconditioning preserves cortical/penumbral tissue in focal ischemia models

In this review article, we first attempted to clarify the IT nomenclature. Various triggers induce cerebral IT; these are mentioned in a separate section discussing the models for IT. This variety among preconditioning triggers indicates that the downstream signaling pathways converge on some common fundamental mechanisms [5], major mechanisms are discussed briefly. A number of tools serve for examination of the efficacy of PC, chief methods are exampled. Lastly, we addressed the challenging issues of IT to encourage further research.

## I. Nomenclature

The nomenclature used in the studies addressing the IT phenomenon is not entirely consistent. In this article, in order to keep with consistency while defining the methodology of the IT experiments and to provide ease for reading, the following terms will be used according to the definitions given:

### PC

The stimulus or the method applied in an experiment that triggers IT in the brain.

### Ischemic preconditioning (IPC)

Method of PC by inducing either global or focal transient cerebral ischemia. When the PC stimulus is different than ischemia, PC will be named according to the nature of the trigger (e.g., hypoxic-PC, anesthetic-PC, chemical-PC). In the literature, sublethal or priming ischemia have been used as alternative terms to IPC. For subsequent ischemic insult given after PC, the term test ischemia is often used [6,7], among others (final, lethal, reference, or full ischemia). We will prefer to name the subsequent ischemic event as final ischemia.

### IT

Briefly, the protection from final ischemia provided by prior PC refers to as IT. Depending on the media used to study the phenomenon of IT, IT may refer to the cell, tissue, or organ's post-ischemic state wherein, due to previous PC exposure, the response to ischemia is different from one observed without previous PC. In this article therefore, PC and IT define two different (but related) entities and are not used interchangeably.

### Ischemia-tolerant phenotype (Figure 1)

It is the consequence of both pre- and post-ischemic protective responses induced by PC [5]; in other words, it is the resulting phenotype from both PC and final ischemia.

### Latent cerebroprotective phenotype

It determines the status of the cell, tissue, or organ exposed to PC that experiences changes triggered by PC, and it occurs before the application of final ischemia [5]. Hence, the latent cerebroprotective phenotype differs from the ischemia-tolerant phenotype by the lack of exposure to final ischemia (Figure 1).

### Cerebroprotection or protection

With the better understanding of the concept of neurovascular unit (i.e., the contribution of glial and vascular endothelial cells and their interactions with neurons in physiological and pathological conditions), researchers' attention shifted from neurons towards cerebrum. Hence, instead of "neuroprotection", we prefer to use "cerebroprotection", which covers not only neurons but all the cerebral cell populations experiencing IT. To interpret IT afforded on single cell type (hippocampal CA1 neurons in global ischemic models or type of cell slice used in vitro study), the protective effect provided by PC will be discussed as "protection".

## II. Two phases of IT

Preconditioning induces two phases of IT with different temporal profiles and, to some extent, with different mechanisms of protection: early and delayed IT (Figure 1); the latter plays the major role in the brain. Early IT is a short-lasting protection induced within minutes of exposure to PC and wanes within hours. In this process, rapid changes in activity and posttranslational modifications of existing proteins are involved, whereas delayed IT requires gene induction and *de novo *protein synthesis, that represent a long-term response through genetic reprogramming [4]. If the final ischemia is induced during the unprotected window, which exists between early and late IT (usually 30 min-1 hour after PC, lasting up to 24 hours), no tolerance occurs (Figure 1).

In the literature, early IT has been termed as the first window of protection [8], rapid IT [5], immediate IT [9], short-term protection [8], classical IT [10], or acute IT [4]. We will prefer to use early IT [10]. Alternative terms for delayed IT are: second window of protection (a term widely used in heart IT studies), classical IT [5], and late IT [11].

## III. Models for IT

**Figure 2:**
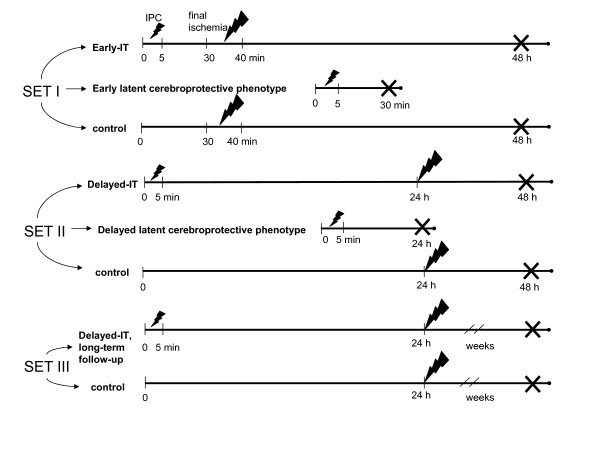
**Exemplary protocol of a series of ischemic tolerance (IT) experiments**. Preconditioning ischemia (IPC) lasts 5 min, final ischemia 10 min. Cross indicates the end of the study where the brains are collected for ex vivo evaluations. Set I experiments evaluate early IT: final ischemia is applied 30 min after IPC (upper row), early ischemia-tolerant phenotype is tested by collecting the brains 25 min after PC, and in the control experiment, final ischemia is induced without prior IPC (lower row). In Set II experiments, delayed IT (upper row, final ischemia induction 24 h after the PC) and delayed ischemia-tolerant phenotype (middle row, brains are collected 24 h after PC) are investigated, along with a control experiment (lower row). Set III experiments address long-lasting effects of delayed-IT, with a follow-up lasting several weeks after final ischemia.

Study setups for investigating potential phenotypes induced by PC are exampled in Figure 2. A summary of the available rodent models of IT is included in the following link as Additional File 1.

## Global-Global

Animal models of global cerebral ischemia are designed to mimic cardiac arrest in humans. Global-global IT models include different durations of transient global ischemia as PC and final insult.

### Four-vessel occlusion

Rat forebrain ischemia experiments necessitate the occlusion of both posterior and anterior blood circulation of the brain. Four-vessel occlusion was originally described by Pulsinelli et al. [12] as a two-stage procedure wherein first both vertebral arteries are permanently closed and the following day, both carotid arteries are occluded. The model has been modified later by the same authors [13] and others [14]. In rats, delayed IT was provided by 3 [15] and 5 min [16] of four-vessel occlusion to 6 to 20 min of final global ischemia [15-17]. Appropriate durations of PC and the interval between PC and final ischemia in this scenario have been studied by monitoring postischemic Hsp72 protein expression as a marker of IT [18]. Neither 3, nor 8 min of IPC induced sufficient synthesis of Hsp72. Once PC was fixed to 4 to 5 min, the minimum 2 days of interval was required between PC and final ischemia.

### Two-vessel occlusion and hypotension

This model, originally described by Smith et al. [19] in the rat, includes bilateral carotid artery occlusion and systemic hypotension induced by withdrawal of arterial blood. Compared to four-vessel occlusion, it is less invasive and more reproducible, as the depth of ischemia depends on hypotension, rather than on surgical attenuation of collateral perfusion [20]. PC by 2 to 3 min of two-vessel occlusion induces delayed IT in rats to 5 to 10 min of final global ischemia [21-23]. Early IT was also achieved in this model [24].

### Two-vessel occlusion in gerbils

The gerbil mostly lacks a functioning circle of Willis [25], a short period of bilateral occlusion of carotid arteries (3-5 min) therefore leads to severe damage in CA1 pyramidal neurons [26,27]. First introduced by Kitagawa et al. [3], PC by single 2-min bilateral carotid occlusion (or two times), 1 to 7 days before final ischemia in the gerbil brain has been a well-standardized method to study IT [28-30]. A disadvantage of the gerbil two-vessel occlusion model is that the severity of forebrain ischemia is highly influenced by the anatomical variations, which are not seldom [31]. Here, we should note that thresholds for severity (i.e., duration of the bilateral carotid occlusion), differentiating the outcome as either PC or final insult, are in a narrow scale. To overcome this issue and ensure a better control over the ischemia severity, Abe and colleagues [7] provided a useful modification of the model. By monitoring depolarizations, they largely eliminated the variability of the ischemia and IT. This approach later was introduced in a rat global-global IT model using four-vessel occlusion [32].

### Two-vessel occlusion in mice

This method, borrowed from its equivalent in gerbils, may induce reproducible striatal injury in mice [33]. For a delayed IT paradigm, Wu et al. applied 6 min of two-vessel occlusion as PC and 18 min of bilateral carotid artery occlusion as final ischemia in C57BL/6 mice [34]. As this strain is a common subject of transgenic technology, the model proved useful for investigating the molecular mechanisms of IT in gene-modified mice. In a such scenario, a much longer two-vessel occlusion period (20 min) has induced delayed IT [35].

## Focal-Focal

### Transient focal-permanent focal

Transient occlusion of the middle cerebral artery (MCA) by intraluminal insertion of a nylon monofilament, which was originally described by Koizumi et al. [36] and modified by others [37], is the most common model to induce focal cerebral ischemia in rats [38-41] and also available in mice [42-45]. This method was introduced first time in a rat IT experiment, applying 10 min of transient MCA occlusion (tMCAO) as the PC stimulus and permanent MCAO as the final ischemia [46]. Authors evaluated IT phenomenon with several reperfusion periods between IPC and final ischemia and showed that ischemic lesions involving both cortex and basal ganglia could be reduced when final ischemia was applied 1, 2, and 7 days after PC, but not 2, 6, and 12 hours or 14 and 21 days after PC. This model was applied successfully by others to obtain delayed IT [47,48]. Repeated brief transient ischemia regimen was also proved as a preconditioning paradigm inducing early IT in mice subjected to permanent focal ischemia [49,50].

### Transient focal-transient focal

One [51,52] or 3 times of 10 min transient focal cerebral ischemia protects from subsequent 120 min of tMCAO in rats [53-55]. Shorter durations (2 and 3 min) of tMCAO were severe enough to induce delayed IT, but did not provide early IT to transient ischemia [56,57]. Transient focal-focal IT paradigm induced IT also in mice and spontaneously hypertensive rats [58,59]. A recent mouse model of delayed-IT involves 2 periods of 5-min tMCAO as the PC method, against 90-min tMCAO applied in 3 days, but not in 2 or 4 days [6].

### Global-Focal

Brief global ischemia can protect from both subsequent transient and permanent focal ischemia [60,61].

## Focal-Global

Brief unilateral occlusion of the MCA induced significant protection from global ischemia in both gerbils [62] and rats [63]. Interestingly, transient (20 min) occlusion of the distal MCA protected only ipsilateral parietal cortex of the rat from global ischemia (10 min) [64].

## Cross-Tolerance

Cross-tolerance is tolerance to ischemia provided by miscellaneous noxious stimuli, rather than ischemia. These differ greatly in nature, nevertheless, because of a common reason (most likely by inducing genetic reprogramming), all furnish IT. Examples of cross-tolerance in the scenario of transient focal cerebral ischemia are provided in Table 2.

**Table 2:** Amount of histological protection afforded by preconditioning in selected studies of focal cerebral ischemia

	Protection* (%)	Follow-up**	Ref.
**Focal-focal, in rats**			
15 min MCAO - 72 h later pMCAO	41	24 h	[47]
10 min MCAO - 72 h later 60 min tMCAO	44	7 d	[59]
3 min tMCAO - 72 h later 60 min tMCAO	35	24 h	[57]
**Focal-focal, in mice**			
3 × 5 min tMCAO - 30 min later pMCAO	23	24 h	[50]
3 × 5 min tMCAO - 30 min later 60 min tMCAO	32	24 h	[49]
15 min MCAO - 72 h later 45 min tMCAO	70	24 h	[58]
**LPS**			
LPS 0.5 mg/kg - 72 h later 60 min tMCAO in rats	35	24 h	[98]
**Hypoxia**			
11% oxygen for 2 h - 48 h later 90 min tMCAO in mice	46-64	24 h	[66]
**Anestesia**			
Isoflurane 1.4% for 3 h - 0, 12, and 24 h later pMCAO in rats	31-35	4 d	[113]
Halothane 1.2% for 3 h - 24 h later pMCAO in rats	35	4 d	[113]
**Spreading depression**			
KCl application - 4 days later 120 min tMCAO in rats	43	4 d	[191]
**Hyperbaric oxygen**			
100% oxygen for 1 h, 5 days-24 h later pMCAO in mice	27	24 h	[69]

Ref, references; *Reduction in the ischemic damage due to preconditioning; **Time-point of the histopathological analysis after final ischemia

### Hypoxia

Exposure of neonatal rats to 8% oxygen for 3 hours provides cerebroprotection from a combined hypoxia/ischemia model [65] and also from both transient and permanent focal cerebral ischemia [66,67]. Varying hypoxia durations (1, 3, or 6 hours) result in similar extent of protection, but when the interval between hypoxia and final ischemia exceeds 72 hours, IT abolishes [67].

### Hyperbaric oxygen

Hyperbaric oxygen was found protective from subsequent global ischemia in gerbils [68] and from permanent focal ischemia in SV129 mice [69], whereas it did not induce IT to transient focal ischemia in these mice [69]. Rats were protected from transient ischemia by hyperbaric oxygen PC, but they were not protected from permanent ischemia [70]. Repeated hyperbaric oxygen application seems to induce IT to global ischemia in the rat brain for a shorter period than 72 h [71].

### Hyperthermia

In rodent experiments, indirect brain temperature can be measured with a probe placed under the temporal muscle and can be maintained at a desired level by heaters allowing feedback adjustments. Chopp et al. first time observed the PC effect of hyperthermia in rats subjected to global ischemia [72]. Hyperthermia was protected as well neonatal rats from hypoxia/ischemia [73].

### Hypothermia

The hypothermic-PC has been described in a rat model of focal transient ischemia [74] and later was studied systematically in order to define the optimal depth, duration, and the method of application (global versus focal hypothermia) [75]. The extent of protection was dependent on the depth and duration of the hypothermia, focal cooling being as effective as systemic cooling. Although the deeper the hypothermia, the bigger the IT response was, mild to moderate levels of hypothermia, which are safe in humans [76], were efficient as well. This may encourage clinicians to test hypothermia as a preconditioning strategy, for instance before vascular surgical interventions with high risk of ischemic events.

### Spreading depression

Leão's spreading depression is a generalized and stereotyped response of the cerebral cortex to a variety of noxious stimuli and is characterized by a slowly moving, transient, and reversible depression of cortical electrical activity that spreads like ripples in a pond; these waves, from the site of onset, spread usually to the whole cortex of the ipsilateral brain hemisphere with a speed of 2 to 5 mm per minute [77]. Topical application of high concentration of potassium chloride onto the cortex induces spreading depression that repetitively extends from the sites of increased extracellular potassium concentration with a frequency of approximately 7/100 min [78]. This method has been an effective PC trigger in both global [79] and focal ischemia models in rats [80-82]. IT induced by spreading depression seems to develop in a delayed manner (in 3-6 days) [83,84] and was shown to persist up to 15 days [85].

### Remote IPC

Limb ischemia by bilateral femoral artery occlusion protects rat from either global ischemia [86] or transient focal ischemia [87]. It can be applied as well repeatedly (5-10 min for 3 times, with 10 minutes intervals in between). This PC approach was successfully tested in humans to induce IT in the heart [88]. Mesenteric artery occlusion for 15 min was protective against bilateral carotid occlusion in mice [89].

### 3-nitropropionic acid (3-NPA)

This is the most extensively studied chemical PC agent, which inhibits oxidative phosphorylation. Intraperitoneal administration of 3-NPA, 72 hours before transient focal ischemia, is a well-established PC trigger for rats [90-93]. Regarding the efficacy of 3-NPA as a PC trigger, some contradictory results came from gerbil models of global ischemia [94-96], but these may be related to the doses used [97].

### Lipopolysaccharide (LPS)

LPS is a cell-wall component of gram-negative bacteria. A small dose provides IT in the brain. This has been proven in a number of experiments including both transient [98,99] and permanent [100,101] focal ischemia models in rats, as well as in a mouse model of transient focal ischemia [102]. With higher doses no PC effect occurs [98].

### Anesthetic-PC

Potential protective effects of anesthetics from an ischemic insult have been known for long time [2] and were well-studied in experimental stroke models as a cerebroprotective strategy (for reviews see [103-105]). Among anesthetics, isoflurane is the most commonly used volatile anesthetic in IT experiments. Different concentrations (0.5-4%) and varying durations (15 min-3 hours) of isoflurane inhalation have been efficient to induce both early [106,107] and delayed IT in vivo [108,109] and in vitro models [110,111]. Among other anesthetics, xenon [112], halothane [113], and sevoflurane [114] may also induce IT in animal models. Anestetic-PC has been proven a promising PC method for heart in humans [115].

### Pharmacological PC

Several clinically available drugs, including estrogen [116], erythromycin [117,118], and erythropoietin [119,120], are capable of inducing IT in animal models. Acetylsalicylic acid [121] and kanamycin [122] were effective as PC agents in vitro.

### Other models for IT

Repeated electroacupuncture [123,124], electrical stimulation of cerebellar fastigial nucleus [125], and dietary restriction [126] protected rats from subsequent transient focal ischemia. In global ischemia rat models, repetitive transcranial magnetic stimulation [127], electroconvulsive shock [128], kainite-induced epileptic seizures [129], and sleep deprivation [130] all have served as PC stimuli.

### *In vitro* models

Neuronal cell culture systems provide an ideal microenvironment to study PC, because they lack a vascular compartment and the environment is easily controlled for confounding factors (e.g., see the fascinating work by Gonzalez-Zulueta et al. [131]). In vitro modeling for ischemia consists of oxygen and glucose deprivation (OGD) in the culture medium, and perhaps the most widely used method is the one described by Goldberg and Choi [132,133]. This model includes the transfer of neocortical cell cultures for several hours to an anaerobic chamber containing a gas mixture of 5% CO2, 10%H2, and 85%N2 (oxygen deprivation), followed by application of a deoxygenated glucose-free medium (glucose deprivation). Organotypic hippocampal slice cultures offer an attractive alternative method, because many aspects of in vivo ischemia, such as delayed death of CA1 neurons and selective vulnerability in response to OGD, can be addressed [134]. Hassen et al. has introduced a new model of IT by isolating hippocampal slices from young rats, to abolish age-dependent resistance to ischemic injury [135]. Also mixed neocortical cultures are available to study IT in vitro [136].

## IV. Methods for detecting IT

In most of the IT studies, the ischemia-tolerant phenotype is addressed with assessments performed after the final ischemia; however, to expose the molecular substrates of latent cerebroprotective phenotype, the tissues should be collected after PC (Figure 2). Studies, which used the latter approach, have been recently reviewed [137]. To increase the relevance to the human condition, IT models should include both histological and functional evaluations. However, these imply more challenges for IT researchers [138], because not always a correlation between these two outcome parameters is present [30].

### Histological techniques

Determining the extent of injury after focal ischemia is relatively simpler than after global ischemia. For this purpose, traditional histological staining techniques, such as hematoxylin-eosine and 2% solution of 2,3,5-triphenyltetrazolium chloride, are often used. Digital camera-based image analysis systems enable lesion area and volume calculations. Ischemic lesion volume is calculated preferably with the correction of edema effect [38,39,139,140]. In IT experiments, reduction in lesion volume due to PC (lesion size in the näive brain -- lesion size in the preconditioned brain) can be calculated as a percent ratio to the lesion size in the näive brain (Table 2). In global ischemia models, ischemic damage is assessed in hippocampal sections stained with toluidine blue [141], cresyl violet [34,94], or thionin [142] by counting CA1 neurons, which are highly susceptible to global ischemia and easy to quantify due to their laminar distribution and large size [138]. Protection due to PC can be reported as the percentage of preserved healthy hippocampal CA1 neurons or number of viable CA1 neurons [7,30,129]. In vitro models of IT use cellular injury assessments, such as lactate dehydrogenase assay [111,143].

### Functional assessment

Gross measures of sensorimotor abilities are available for rodents [37,144], and were introduced in IT experiments [46,47,97,107]. However, in these species, gross sensorimotor deficits tend to recover rapidly. That is, more complex tests are needed, especially if outcome is assessed in long-term. A number of somatosensory tests (e.g. limb placing, beam walking, grid walking, rotarod) are available to apply in focal ischemia rat models [145]. In global ischemia models, tests of learning ability, and working and reference memory are particularly useful [138,146].

### Lesion evaluation by magnetic resonance imaging (MRI)

MRI technology allows for temporal and spatial monitoring of ischemic lesion and enables to conduct longitudinal studies [147-151]. Besides requiring anesthesia, MRI is risk-free for experimental animals. First MRI-based lesion evaluation in an experimental IT study was reported by Mullins et al. [152]. In a delayed IT model (focal--focal ischemia), rats were imaged 24 and 72 hours after final ischemia. Interestingly, lesion reduction due to PC was greater at 72 hours (70%) compared to that at 24 hours (53%). Authors concluded that 24 hours post-ischemia, which is a common time-point for lesion evaluation in experimental stroke studies [153-155], may not be the best time-point for experimental IT studies. Furuya et al., imaged rats serially (at 6 and 24 hours and 4, 7, and 14 days), following a delayed IT paradigm (LPC-PC--focal ischemia) [156]. They evaluated whether decreased lesion size due to PC would increase in long-term. No delayed lesion progression was found.

### Means for depicting the mechanisms of IT

Immmunohistochemistry is widely applied in IT research and serves to evaluate tissue alterations by means of antigen-antibody interactions. In situ hybridization and Western blotting techniques are applied to examine the effect of PC on the investigated protein's mRNA expression and abundance [141]. DNA microarray technology, which allows quantification and differential expression of thousands of genes simultaneously, has been used to investigate global changes occurring between ischemia-tolerant and näive brains (see below "genomic reprogramming"). Real-time PCR can be used for confirmation of the selected genes, which found upregulated by microarray analysis [157]; proteomics may provide supplemental insights [158]. Alterations in neurotransmitter receptor density can be evaluated by quantitative in vitro receptor autoradiography [159,160]. Autoradiographical methods may show changes in the global protein status [29,161].

Showing attenuation or abolishment of IT by pharmacological inhibition of a molecule before or after PC stimulus proves a robust approach. With this approach, necessary or mandatory components of IT can be explored [162,163]. Complementary information may come from genetically modified animals by proving abolishment of IT in mutants lacking a functional molecule or protein and showing reestablishment of IT in rescue experiments [164]. Maintenance of IT, despite pharmacological inhibition of a molecule of interest or despite the lack of this molecule in the mutant mouse, may rule out the hypothetic causative role for the investigated molecule in the acquisition of IT [35,84,163]. However, it should be noted that, the main effectors of IT can be model- or trigger-specific that, for instance, a specific molecule proven mandatory for hypoxia-induced IT in the rat brain [165] may not necessarily be required in OGD-induced IT in vitro [166].

## V. Mechanisms of IT

IT is achieved by the attenuation of broad categories of injury-inducing mechanisms, including excitotoxicity, ion and pH imbalance, oxidative and nitrosative stress, metabolic dysfunction, inflammation, and apoptotic cell death. Additionally, innate survival mechanisms and enhanced endogenous repair mechanisms are involved [5]. Preservation of energy metabolism and mitochondrial functions during the ischemic event is improved [167,168]. Our knowledge on the underlying mechanisms of cerebral IT is yet patchy. Additionally, different mechanisms may dominate different models. Here, we will review only the major molecular aspects contributing to delayed cerebral IT. The mechanisms of early IT will be discussed separately at the end of this section. Readers seeking for more comprehensive information should consult the recent excellent review of Obrenovitch [169] as well as its antecedents [9,133,167].

### Hypoxia-inducible factor-1 (HIF-1)

Among several transcription factors sensitive to regulation by hypoxia/ischemia, HIF isoforms have gained the most experimental support [5]. HIF-1 proteins are increased in the brain in the setting of hypoxia resistance [170] and hypoxic PC [67]. Pharmacological activators of HIF-1 (deferoxamine or cobalt chloride) promote PC in hypoxia/ischemia model in neonatal rats [170]. Over the past decade, the signalling pathways involved in HIF-1 activation have been deciphered in detail [171]. Briefly, hypoxia stabilizes alfa subunit of HIF-1, which enters the nucleus in a dimerized form and results in the induction of HIF target genes. Several HIF target genes contribute protection from ischemia [67,172,173], and their products involve in wide range of adaptive and pro-survival events, including cellular metabolism, proliferation, vascularization, iron homeostasis, and glucose metabolism [5,133].

### Protein kinase C (PKC)

The role of protein kinase C in mediating stroke injury has been reviewed recently [174]. There are 10 isozymes in the PKC family. Previously, PKC was thought not to have a role in IT phenomenon, because blockage of PKC did not prevent IT [175], and PKC activation did not induce IT [176]. However, accumulating data suggest opposite roles for different PKC isozymes in the brain: γPKC contributes in ischemic cell death in organotypic hippocampal cell cultures, and NMDA triggered IT models require εPKC translocation [177]. Even though non-selective activation of PKC does not induce IT, specific εPKC activation leads to IT [143,177]. It seems that adenosine-mediated activation of εPKC and subsequent signal transduction pathways through MAPK-K, ERK [178], and cyclooxygenase-2 induction are involved in IT [143].

### Anti-excitotoxic mechanisms, NMDA, and calcium

Exogenous application of NMDA or glutamate alone suffice to induce ischemia resistance in cell cultures, and NMDA receptor blockade during preconditioning eliminates IT both in vitro [179] and in vivo [180,181]. Specific AMPA or kainate receptor blockade do not eliminate or only partially attenuates IT [131,180]. Contradictory findings exist, however [136,182]. In gerbils, IPC increased inhibitory γ-amino butyric acid A (GABA_A_) receptor binding in hippocampus, whereas final ischemia did not [160]. Moreover, microdialysis experiments revealed a temporary increase in GABA release in preconditioned rat hippocampus early after final ischemia, with a decrease in glutamate concentration [21]. Thus, anti-excitotoxic mechanism induced in ischemia-tolerant state in global ischemia models involve a shift between inhibitory and excitatory hippocampal neurotransmission. In vitro, GABA_B _activation operates as a PC trigger [21], but not GABA_A _activation [21,183]. Regarding the role of Ca^2+ ^in IT, it seems not always mandatory [184], but chelation of Ca^2+ ^before and during both OGD- and NMDA-PC prevents IT in vitro [177].

### Adenosine and ATP-sensitive K^+ ^(K_ATP_) channels

Adenosine, an ischemia-induced degradation product of ATP, activates A_1 _receptors, which leads to a cascade of signaling events including K_ATP_channels. This cascade results in increased resistance to subsequent ischemic damage [185]. The general role of K_ATP _channels, which are named for the inhibitory effect of ATP reducing channel opening probability, is to set membrane potential according to its metabolic state by sensing intracellular nucleotide concentrations [186]. Plasma membrane K_ATP _channels are found widely throughout the brain [186]. The mandatory role of K_ATP _channels for acquisition of IT was demonstrated in a rat delayed IT model (global-global) [187] and in vitro [188]. Interestingly, early IT is also blocked by pharmacological inhibition of K_ATP _channels in vitro [176]. Opening of K_ATP _channels is thought to relate to adenosine A1 receptor activation. Both specific and nonspecific adenosine A1 receptor antagonists attenuate or cancel the IT phenomenon [187,189,190]. However, SUR1-containing K_ATP _channels seem not to be involved in IPC [35] and in spreading depression-PC model in rats and inhibition of K_ATP _channels did not block IT [191]. Some authors emphasize a more pronounced role for mitochondrial K_ATP _channel in IT [169,190].

### Nitric oxide (NO)

NO is one of the most extensively studied molecules in IT experiments (for reviews see [192,193]). Data suggest that generation of NO is crucial for the induction of IT, as a dependence on endothelial NO synthase (eNOS), but not on the neuronal NOS (nNOS) in newborn rats subjected to hypoxic-PC [165]. Whereas, nNOS was required to induce tolerance in vitro [166]. OGD tolerance in cortical cell cultures occurred via the activation of the Ras/extracellular signal-regulated kinase cascade by NO [131]. Atochin's early IT model proved an indispensable role for both eNOS and nNOS [50]. Puisieux et al. used a delayed IT (focal-focal) model in adult rats and showed no effect of NOS blockade on IT, but when the PC stimulus was LPS, IT was abolished by NOS inhibition [194]. Inducible NOS (iNOS) lacking mice experience no IT [164] and iNOS inhibition may nullify delayed IT to permanent focal ischemia, that otherwise follows isoflurane- or halotane-PC [113].

### Anti-inflammatory mechanisms

Interleukin-1 (IL-1) and tumor necrosis factor-α (TNF-α) are implicated in IT induction: both cytokines are found increased in ischemic-tolerant state, both act as PC trigger when administered systemically, and their inhibition or lack significantly attenuate or block IT [195-198]. Pradillo et. al. explored the involvement of the TNF-α/nuclear factor-κB (NF-κB) signal transduction pathway in IT [48]. This pathway includes at least 131 interactors [199]. Activation of NF-κB is involved in IT in several models [200,201], likely via the induction of neuroprotective gene products, such as manganese superoxide dismutase and Bcl-2 [9]. Preconditioning with ligands of toll-like receptors 4 and 9 may alter innate inflammatory responses to ischemia by causing an initial activation of inflammatory mediators followed by a burst of inflammation inhibitors [202].

### Anti-apoptotic mechanisms

PC blocks enhanced phosphorylation occurring after ischemia [9]. On the other hand, phosphorylation of transcriptional factors can induce long-term changes by regulating the expression of genes. IT is also characterized by reduced apoptosis [5,142]. Phosphaphatidylinositol 3-kinase/Akt pathway seems to act in two ways: 1) in relation to anti-apoptotic mechanisms and 2) by activating NFkB. In vitro, p21 Ras is required and sufficient to induce IT and Ras/Erk pathway is activated through NMDA receptor and NO production [131]. However, increasing evidences support the existence of a link between Akt activation and anti-apoptosis in IT [157,203-205], perhaps more persistently in penumbral regions in focal IT models [206]. Anti-apoptotic mechanisms induced by PC are several: induction of Bcl-2, reductions in caspase-3 synthesis and p-53 activation, and reductions in mitochondrial cytochrome c [9,185].

### Genomic reprogramming

With the contribution of DNA microarray analysis method to IT research, we gained a better understanding of the preconditioned brain on the genetic level. In 2003, Stenzel-Poore and colleagues published a study, a cornerstone in the field, which introduced the concept of "genomic reprogramming" defining the altered transcriptional response of the ischemia-tolerant brain [207]. Followed by others [208,209], profiled the genetics of IT induced by IPC in rats were profiled. In the setting of IT, overall transcriptional response to injury was found altered as downregulation, which was strikingly different from that in the naïve brain's postischemic transcriptome. Suppression of gene expression in the ischemia-tolerant state was not simply the lack of response to injurious insult, but rather a reprogramming of the genetic response to ischemia [210]. Most of the genes suppressed are involved in the pathways that regulate metabolism, molecular transport, or cell-cycle control. Genomic transcriptional profile shows a substantial difference also between latent cerebroprotective and ischemia-tolerant states. None but one of the differentially regulated genes compared to healthy hemisphere are in common [208]; however, in both states, overall response is downregulation of genes involved in metabolism and transport/synaptic transmission. Using GeneChip analysis, Dhodda et al. evaluated temporal changes in gene expression after IPC in spontaneously hypertensive rats [158]. At the time-points studied (3, 6, 12, 24, and 72 h after PC), overall 40 transcripts were found up-regulated, among which 30 transcripts were overexpressed at all time-points, and the six HSP70 transcripts showed the highest increase. Other major families of transcripts, which were upregulated during PC, were those that control signal transduction, transcription, ionic homeostasis, and plasticity. Moreover, transcripts that showed upregulation after ischemia in näive brains were not found upregulated in ischemia-tolerant brains [158].

Gene expression response to hypoxic-PC was also studied [173]. As early as 1 hour after hypoxia, but at a greater extent at 6 hours, expression of many genes, which are regulated by HIF-1, were increased. Compared to näive ischemic brains, in the ischemia-tolerant brains preconditioned with hypoxia, several genes were differentially upregulated. Genes with decreased expression in näive ischemic brains were no longer or only to a small degree underexpressed in ischemia-tolerant brains.

Genetic response to hyperbaric oxygen-PC was studied in the rat, in the latent cerebroprotective state (at 6, 12, 24 after PC) [71]. Most of the differential regulations, including overexpression of genes and proteins related to neurotrophin and inflammatory-immune system, occurred around 12 and 24 hours. Genetic reprogramming was described as well for IT induced by erythromycin [118].

### Mechanisms of early IT

In vivo models demonstrating early IT in the brain are limited: global-global model in the rat [24], and focal-focal model both in the rat [211], and mouse [49,50], and anesthetic-PC against focal permanent ischemia in the rat [113].

The molecular mediators of early IT are little known. Changes in membrane channel activity and posttranslational modifications of existing proteins are among the few, which are well-described. Roles for adenosine receptor in vivo [211] and for K_ATP _channel in vitro were also explored [176]. Several immediate-early genes (c-fos, c-jun), growth factors (brain-derived neurotrophic factor, nerve growth factor), and heat shock protein 70 were overexpressed during early latent ischemia-tolerant state [212]. According to Kariko et al. [213], during early tolerance, production of proinflammatory cytokines are suppressed, whereas in delayed tolerance, production of the very same cytokines are induced.

## VI. Open Issues and Challenging Features of IT

Several specific questions arise by an overview of past IT experiments. The nature of the PC stimulus and the duration of the interval between PC and final ischemia are among the main parameters that may affect the results. The strain and gender of the experimental animal are additional sources of variability, as we are familiar from stroke experiments. Therefore, findings of an experimental IT study should be interpreted considering the following issues.

### Trigger-dependent differences

Experimental data amounted for the last 20 years clearly demonstrated that IT can be afforded in animals by miscellaneous PC triggers. Thus, one can both easily and reasonably make the following assumption: diverse PC are sharing a common or overlapping pathway. As discussed above, a number of effector mechanisms confer ischemia-tolerant phenotype, and recently, genetic reprogramming was proposed as the underlying common process set into motion by these mechanisms [207,208]. Below, we will have a closer look to studies comparing the mechanistic or molecular features of IT triggered by different PC stimuli.

IPC versus LPS-PC was compared in a transient ischemia rat model and found inducing similar degree of protection (35% reduction in infarct volume) [194]. An interesting finding was that NO synthase inhibition abolished the protective effect of LPS, but not of IPC. IPC induced the expression of heat shock protein 70 in the cerebral cortex, but LPS did not. Recently, ischemia-tolerant phenotypes induced by two well-known preconditioning stimuli -LPS and transient focal ischemia- have been evaluated from the genetic aspect [209]. Authors disclosed that a substantial subset of regulated genes were unique to each PC stimulus. In case of IPC, mainly metabolism and channel/transport-related genes were suppressed; whereas, LPS-PC induced expression of pro-inflammatory molecules and suppressed those genes related to deleterious inflammatory reactions. However, suppression of Toll-like receptor-mediated inflammation is a common mechanism triggered by both PC triggers [213]. Another comparative study of different PC stimuli (IPC and chemical PC with 3-NPA) addressed cytokine mRNA expression after final ischemia [214]. Both PC strategies exerted very similar effects on proinflammatory and cytotoxic cytokine expressions. Later, same authors studied the expression of nerve growth factor separately with IPC and 3-NPA-PC paradigms [215]. Neither trigger showed any effect on nerve growth factor expression, which in another study was found increased by PC with brief global ischemia in both early and delayed IT [212].

### Intermodel differences

In focal-global IT paradigm, PC may confer IT in neurons outside the primary area subjected to IPC that is in proximity, but not in the further regions such as contralateral hippocampus [62]. Similar IT paradigm in rats resulted in bilateral protection of hippocampi, however [63]. A functional direct pathway from the entorhinal cortex to both hippocampi was suggested to reflect the changes afforded by IPC to both hemispheres [63]. In the global-global IT paradigm, c-fos expression during the tolerant state was found specific to the cell type [216], which may explain selectivity of IT induction to certain brain areas.

Prass et al. studied the confounding effects of strain and reperfusion on the IT phenomenon [69]. Hyperbaric oxygen was applied as PC stimulus to two common background strains for knockout mice, SV129 and C57BL/6. Final ischemia was either permanent or transient focal ischemia. In SV129 mice, PC induced tolerance to permanent ischemia but not to transient ischemia. In C57BL/6 mice, IT did not occur at all. Consequently, questions to answer with further study are: 1. For what reasons the very same trigger induced IT in a strain but not in another, and 2) Can reperfusion nullify the protection afforded by PC?

### Gender

Female rats sustain smaller infarcts after MCAO than males [217] and estrogen is neuroprotective in ovariectomized females and in males subjected to ischemic stroke [218]. Data from heart IT experiments show a clear gender-dependency of the IT phenomenon [219,220], this issue seems valid also in cerebral IT. Estrogen provided IT in a model of hippocampal organotypic slice culture, which was generated from neonatal female rats [116], and isoflurane induced IT only in male mice and increased the infarction in young female mice [221].

### Age

IT phenomenon is preserved in aged animals [222], but may not be as effective as it is in young animals. This aspect was tested with a global-global IT paradigm applied in 4- and 24-month-old rats [223]. The degree of protection due to PC was significantly diminished in aged rats compared to young rats. A retrospective clinical study indicated that IT may not be occurring in the elderly, aged around 75 [224].

### Repeated PC

Cumulative injurious effect of repeated cerebral ischemia is a well-known phenomenon. For example, three periods of 5-min forebrain ischemia, induced at 1-hour intervals, result in more extensive brain injury than one single episode of 15-min ischemia in gerbils [225]. However, if PC insults are applied repeatedly, a larger IT response may be gained. This was tested in a mice model of early IT, in which animals underwent either single or 3 episodes of 5-min focal cerebral ischemia, 30 min before permanent ischemia [49]. Only repeated insults conferred IT, the single brief ischemia was insufficient to induce IT. Similarly, a single episode of 2 min OGD is under the threshold to act as a PC stimulus, but four times repeated 2 min of OGD show a cumulative effect and protects from subsequent injurious insult [226]. Hyperbaric oxygen-PC, when applied singly or repeatedly, provide similar degree of protection from transient focal ischemia (63% vs 73% lesion reduction) [227], perhaps this is the maximum affordable protection by hyperbaric oxygen. In a clinical study however, anesthetic-PC with a single application induced no IT in the heart, whereas repeated application did [115]. In the pig heart, PC by repetitive ischemic insults was shown to induce a different set of genetic regulations from those induced with PC with single ischemic episode [228]. A corresponding study in cerebral IT is needed.

### "Sublethality" of PC

Although PC is defined as a sublethal stimulus, which per se causes no injury, several studies used relatively severe focal ischemia as the PC trigger and were able to induce IT, despite the injurious nature of the PC itself [63,64,190]. As pointed out by Sommer [137], with extended follow-up after the PC insult, some injury or structural changes can be detected. Therefore, it is suggested that PC is postponing these changes [137]. If that holds true, in the long-term, näive ischemic brains and IT experienced brains may have similar outcomes. This issue is discussed next.

### IT and long-term effects

Early IT is a short-lasting phenomenon, its protection vanishes around 7 days [24]. In delayed IT models, protection lasts longer and tends to decline after 30-60 days. Ohno et al. applied a global-global IT model to rats [229] and showed that improvement in learning and memory due to IPC was preserved up to 3 weeks. Protective effects of spreading depression-PC and LPS-PC sustained up to 14 days [84,156]. Ma et al. found a sustained improvement in neurological scores up to 30 days in xenon-preconditioned neonatal mice subjected to global ischemia [112], a similar finding was reported with a focal-focal IT model in rats [51]. In global-global IT models, histological protection is longer preserved in rats (up to 90 days) than in gerbils (up to 60 days) [30,32,222,230]. Optimizing time interval between PC and final ischemia, together with the optimization of the PC stimulus (single or repetitive application) and the severity of final ischemic insult, may result in long-term preservation of protection achieved by PC [32], on which increased neurogenesis after PC [231] may have a potential role.

## VII. Clinical Aspects

To date, a body of evidence, which supports the hypothesis that TIAs may confer IT in humans, exists. In a retrospective study, preceding TIA was found to be associated with less-severe stroke on admission and improved outcome on follow-up, compared to stroke patients without preceding TIA [232]. Another retrospective case-control study, found no evidence of PC by TIA in baseline neurological scores, but favorable outcome was associated with the presence of TIA [233]. This study presented "potentially preconditioning" TIA characteristics as: 0-7 day interval between TIA and stroke, 2 or 3 times repeated TIA, and TIA with <20 min duration. Moncayo et al. reported a cohort of 65 patients with acute anterior circulation stroke, among whom those with previous TIAs (lasting less than 20 minutes), had a more favorable outcome than those without [234]. Apparently, duration of TIA should be taken into account while evaluating whether IT exists in humans or not [235]. An MRI study provided the tissue evidence for TIA-induced tolerance to ischemic stroke [236]. Ischemic lesions tended to be smaller on the baseline images and final infarct volumes were smaller in stroke patients with prior TIA than in those without.

Although these findings strongly suggest TIA as the clinical correlate of IPC, other explanations for milder strokes after preceding TIA must be considered. In these patients, a carotid disease with slowly progressing stenosis, which improves collateral circulation may predominate [237]. Another point is that, patients with cardioembolic stoke have lower incidence of TIA than those with atherosclerotic vascular disease, and probably because of larger-sized emboli they sustain larger infarcts and poorer outcome [238].

Several clinical conditions may benefit from strategies using principles of ischemic tolerance, as discussed elegantly by Dirnagl et al. in a recent review article on cerebral IT [239]. Mediators of IT could be used as biochemical markers of IT in stroke patients. Castillo et al. tested this hypothesis by evaluating blood levels of TNF-α and IL-6 in acute stroke patients with or without prior ipsilateral TIA [240]. Better outcome was found in patients with TIA, who showed high plasma concentrations of TNF-α and low concentrations of IL-6. Hence, authors proposed the index of TNF-α/IL-6 as a marker of IT phenomenon in humans.

## Conclusions

Experimental IT paradigms investigate the endogenous pathways by which the brain might protect itself from ischemia when geared with an appropriate stimulus. Attempts to elucidate the mechanisms underlying cerebral IT are increasing exponentially, but diversity of models, including PC stimuli, hardens interpretation of the data. In addition, narrow safety margin of PC may prove a limiting factor of the therapeutic utility of PC in clinics. On the other hand, accumulating clinical data suggest that IT might be a clinically relevant phenomenon. Several approaches, including ICP [241], remote-PC by limb ischemia [88], pharmacological-PC with nitroglycerine [242], and anesthetic-PC [115], are tested in clinical trials to protect the heart from cardiovascular interventions with high risk of cardiac ischemic event. Results are promising and give hope that clinical trials of PC to protect brain in situations with a high risk of ischemia can be designed, once PC is proven safe.

## Abbreviations

3-NPA: 3-nitropropionic acid; GABA: γ-amino butyric acid; HIF-1: hypoxia-inducible factor-1; IL-1: interleukin-1; IT: ischemic tolerance; IPC: ischemic preconditioning; K_ATP_, ATP-sensitive K^+^; LPS: lipopolysaccharide; MCA: middle cerebral artery; MCAO: middle cerebral artery occlusion; MRI: magnetic resonance imaging; NO: nitric oxide; nNOS: neuronal NO synthase; iNOS: inducible NOS; eNOS: endothelial NOS; NF-_K_B: nuclear factor _K_B; OGD: oxygen-glucose deprivation; PC: preconditioning; PKC: protein kinase C; TIA: transient ischemic attack; TNF-α: tumor necrosis factor-α; tMCA: transient MCAO.

## Competing interests

The authors declare that they have no competing interests.

## Authors' contributions

Both AD and TT made the conception and design of the manuscript and analysis and interpretation of the data, drafted and revised the manuscript, and have given approval of its final version.

## Supplementary Material

Additional file 1 Models for ischemic tolerance in rodents. Click here for file
